# Whole Genome Gene Expression Meta-Analysis of Inflammatory Bowel Disease Colon Mucosa Demonstrates Lack of Major Differences between Crohn's Disease and Ulcerative Colitis

**DOI:** 10.1371/journal.pone.0056818

**Published:** 2013-02-13

**Authors:** Atle van Beelen Granlund, Arnar Flatberg, Ann E. Østvik, Ignat Drozdov, Bjørn I. Gustafsson, Mark Kidd, Vidar Beisvag, Sverre H. Torp, Helge L. Waldum, Tom Christian Martinsen, Jan Kristian Damås, Terje Espevik, Arne K. Sandvik

**Affiliations:** 1 Centre of Molecular Inflammation Research, Norwegian University of Science and Technology, Trondheim, Norway; 2 Department of Cancer Research and Molecular Medicine, Norwegian University of Science and Technology, Trondheim, Norway; 3 Department of Laboratory Medicine, Norwegian University of Science and Technology, Trondheim, Norway; 4 Department of Gastroenterology and Hepatology, St. Olav’s University Hospital, Trondheim, Norway; 5 Department of Pathology, St. Olav’s University Hospital, Trondheim, Norway; 6 Department of Infectious Diseases, St. Olav’s University Hospital, Trondheim, Norway; 7 Bering Limited, Richmond, United Kingdom; 8 Department of Surgery, Section of Gastroenterology, Yale School of Medicine, New Haven, Connecticut, United States of America; Inserm, France

## Abstract

**Background:**

In inflammatory bowel disease (IBD), genetic susceptibility together with environmental factors disturbs gut homeostasis producing chronic inflammation. The two main IBD subtypes are Ulcerative colitis (UC) and Crohn’s disease (CD). We present the to-date largest microarray gene expression study on IBD encompassing both inflamed and un-inflamed colonic tissue. A meta-analysis including all available, comparable data was used to explore important aspects of IBD inflammation, thereby validating consistent gene expression patterns.

**Methods:**

Colon pinch biopsies from IBD patients were analysed using Illumina whole genome gene expression technology. Differential expression (DE) was identified using LIMMA linear model in the R statistical computing environment. Results were enriched for gene ontology (GO) categories. Sets of genes encoding antimicrobial proteins (AMP) and proteins involved in T helper (Th) cell differentiation were used in the interpretation of the results. All available data sets were analysed using the same methods, and results were compared on a global and focused level as t-scores.

**Results:**

Gene expression in inflamed mucosa from UC and CD are remarkably similar. The meta-analysis confirmed this. The patterns of AMP and Th cell-related gene expression were also very similar, except for *IL23A* which was consistently higher expressed in UC than in CD. Un-inflamed tissue from patients demonstrated minimal differences from healthy controls.

**Conclusions:**

There is no difference in the Th subgroup involvement between UC and CD. Th1/Th17 related expression, with little Th2 differentiation, dominated both diseases. The different *IL23A* expression between UC and CD suggests an IBD subtype specific role. AMPs, previously little studied, are strongly overexpressed in IBD. The presented meta-analysis provides a sound background for further research on IBD pathobiology.

## Introduction

The term inflammatory bowel disease (IBD) mainly covers ulcerative colitis (UC) and Crohn’s disease (CD). IBD is a global health problem, with a reported prevalence as high as 568 and 827 per 100 000 in USA and Europe, respectively[1]. In IBD genetic susceptibility together with environmental factors disturbs intestinal homeostasis, resulting in repeated inflammation-remission cycles. CD can manifest itself anywhere in the gastrointestinal tract, while UC is only seen in the colon with varying length and degree of continuous inflammation extending proximally from the rectum. In UC the inflammation is found in the mucosa, in CD a deeper, often transmural inflammation with fistula formation is seen. Several extraintestinal diseases are associated with IBD, such as skin diseases, seronegative arthritis, uveitis and primary sclerosing cholangitis. Long-standing UC is associated with an increased risk of colorectal cancer. Despite the differences between UC and CD, there are cases where a definite diagnosis cannot be made, resulting in a diagnosis of non-specific colitis.

The coordinated effect of various T helper (Th) subtypes is fundamental to gut homeostasis[2], and UC and CD have been considered different with respect to Th cell activation. In mouse models it was shown that Th1 mechanisms mediated an inflammation similar to CD, and Th2 similar to UC[3]. In later years the concept of Th17 cells has been introduced and it was shown that inflammation previously attributed to Th1 could actually be Th17-driven and that these lymphocytes played an important role in CD[4], [5]. Together with a better understanding of the importance of Treg lymphocytes in controlling inflammation, these discoveries have made it necessary to re-evaluate the Th1/Th2 concept of IBD [6].

Since the emergence of whole genome gene expression analysis (WGGE), efforts have been made to identify the transcriptional regulation underlying IBD. Although nearly a hundred susceptibility SNPs for IBD have been found, there has been limited success in translating gene expression results into hypotheses that aid the understanding and treatment of IBD [7]. Previous work in this field differs greatly. Gene expression technology has evolved fromserial analysis of gene expression (SAGE), to the newest WGGE arrays from e.g. Affymetrix and Illumina. Already in 1997 Heller et al. studied CD using spotted cDNA arrays [8]. In 2000 Dieckgraefe et al. examined colonic mucosa samples using Affymetrix Hum 6000 arrays with a coverage of ∼6500 genes and expressed sequence tags (ESTs). This study identified 74 differentially expressed genes between inflamed UC and normal mucosa, grouping in functional classes such as immunoregulation and tissue regeneration [9]. Following these initial efforts many studies have been carried out, with great variation in the approach to the subject [10]–[25].

The WGGE analyses have identified regulation of several genes involved in processes thought to be of importance for IBD. Wu et al. suggested that genes involved in cell adhesion and polarisation processes were down-regulated in un-inflamed UC [11]. Olsen et al. used a set of genes to create a classification model able to discern un-inflamed UC samples from un-inflamed CD/control samples based on expression levels [12]. By comparing samples from IBD patients refractory to corticosteroids and/or immunosuppression before and after infliximab-treatment, Arijs et al. confirmed previous observations that expression of antimicrobial peptide (AMP) genes is changed in IBD [14], [26], [27]. Other studies have focused on identifying transcriptional regulation that could explain the clinical differences seen between UC and CD. However, the details around the initiation, propagation and maintenance of the chronic inflammation in IBD remain unclear.

When studying gene expression in complex tissues, the choice of sample material is very important. Patient and sample heterogeneity will greatly influence the measured expression levels, often hindering interpretation of the differences between sample groups. In IBD, leukocyte infiltration, Paneth cell metaplasia, crypt hyperplasia and ulceration with loss of epithelial cells are factors that will influence measured gene expression levels. As an example, the increase in α-defensin expression in colonic IBD has been attributed to colonic Paneth cell metaplasia, while the decrease of α-defensin expression in ileal CD has been linked to loss of epithelial tissue, Paneth cell function and *NOD2* status [14], [27]–[29].

Another effect potentially interfering with IBD microarray analysis is the regional variation in gene expression, with both a dichotomous and a more gradually varying gene expression pattern along the colon [30]. This regional variation has been discussed in several earlier IBD gene expression studies. Wu et al. and Costello et al. identified no such gene expression differences due to regional variation [11], [15], In a later study, Noble et al. noted that this was readily identified in healthy controls. It is possible that modest regional variations can be masked when analysing inflamed tissue, but become apparent when un-inflamed biopsies are studied. These observations emphasize the importance of avoiding, or being aware of, confounding effects in the analysis of subtle differences in expression.

In this paper, a microarray-based gene expression analysis of colon pinch biopsies from IBD patients is presented. It is to the best of our knowledge the largest such study undertaken including samples from both inflamed and un-inflamed mucosa from UC and CD patients as well as normal controls. All un-inflamed samples and controls were obtained from the hepatic flexure, minimising any regional effect. The analysis was further supplemented by a meta-analysis of available IBD WGGE data, placing the presented data in the context of previous research on key fields in IBD research; T helper cell activation and antimicrobial peptide expression. The approach used in this work is meant to overcome the challenges of sample size, and heterogeneity in patients and sample material typically seen in gene expression analysis of complex, multifactorial diseases. By exploring the consensus between several data sets for expression related to important aspects of IBD, this analysis serves as a robust validation of suggested hypotheses

## Results and Discussion

### Gene expression analysis – NTNU data

#### Clinical material

All biopsies were assessed as either diseased or normal based on endoscopic findings at time of collection. Final diagnosis was done by histopathological evaluation of H–E stained sections. In cases where there was a discrepancy between endoscopic and histological assessment, the sample was excluded from the analysis. Moreover, 6 patients were excluded after a diagnosis of indeterminate colitis. A summary of sample information is provided in Table 1. Full information on the samples used in the analysis is included in Table S1. The final sample population consisted of 20 healthy controls (N), 37 active ulcerative colitis (UC), 7 active Crohn’s disease (CD), 44 un-inflamed ulcerative colitis (UCU) and 19 un-inflamed Crohn’s disease (CDU) samples. UCU sample group was further divided in two groups based on patient history. Samples originating from patients where sampling area (hepatic flexure) had previously been diagnosed as inflamed (UCUi, 11), and samples where no inflammation of the sample area ever had been observed (UCUu, 23).

**Table 1:** Summary of sample information.

	N	CD	UC	CDU	UCU
Number of subjects	20	7	37	19	44
Age, median years (range)	45 (19–71)	31(20–41)	38(19–72)	39(20–61)	45 (21–71)
Female / Male	9/11	2/5	22/15	6/13	24/20
Duration of disease, median years (range)	NA	7 (1–12)	9 (0–40)	6 (0–28)	13 (0–40)
5-ASA/S-ASA (%)	0	2 (29)	23 (62)	6 (32)	27 (61)
Systemic corticosteroids (%)	0	2(29)	9(26)	8 (42)	4(9)

Each column summarizes characteristics for all patients contributing with samples to the corresponding sample groups. 5-ASA – 5-aminosalicylic acid. S-ASA – sulphasalazine. Sample groups are abbreviated N for normal controls, CD for Crohn’s disease, UC for ulcerative colitis, CDU for un-inflamed Crohn’s disease and UCU for un-inflamed ulcerative colitis.

#### Microarray gene expression analysis

**Figure 1:**
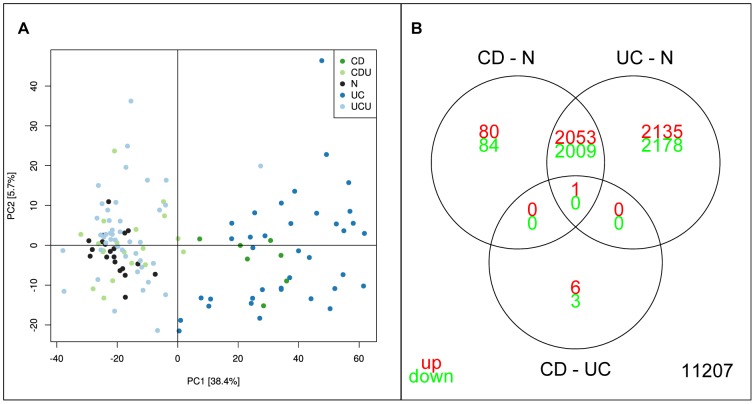
Initial analysis of NTNU data set. A: PCA analysis of NTNU data set. Each point represents one sample, with colour indicating sample groups as described in figure legend. Axis indicate % of total variance explained in each component. B: Venn diagram illustrating the relationship between analyses of sample groups UC and CD directly and using N as common reference. Numbers indicate significant genes (corrected p-val<0.05).

Data analyses were performed using Bioconductor for the R software environment (http:www.r-project.org)[31]. Two samples, 226F and 115F, were analysed 8 times on separate chips as technical controls, showing good correlation between technical replicates (226F r2 = 0.989, 115F r2 = 0.992). In the final analysis the replicate closes to median of all replicates was used. Principal component analysis (PCA) analysis identified inflammation status as the dominating variation in the dataset, separating CD/UC samples from N/UCU/CDU as shown in Figure 1A. There was no apparent separation between UC and CD in the PCA analysis. T-testing identified 4187/4189 significantly down/up-regulated genes for inflamed tissue from UC patients (UC), and 2093/2134 for inflamed tissue from CD patients (CD). For un-inflamed tissue (UCU and CDU), a very low number of differentially expressed genes were found (0/3 CDU, 0/0 UCU). Similarly to what was seen for the collected group, no differential expression was detected for the groups UCUi and UCUu when contrasted against normal controls (data not shown). The latter observation stands in contrast to recent finds reported by Planell et al, demonstrating several thousand genes as differentially expressed between UCUi and N[32]. This discrepancy is thought to arise due to differences in sample inclusion criteria. The complete table of results from the analysis is available in Table S2.

A Venn diagram illustrating the relationship between genes differentially expressed in UC and CD is given in Figure 1B. Using normal samples as common reference identified 4063 differentially expressed genes as common for CD and UC, with 4313 unique for UC and 164 unique for CD. This was in contrast to an analysis of CD vs. UC without use of common reference which identifies only 10 differentially expressed genes, of which 7 were included in the two previous analyses. This discrepancy could arise as a result of the imbalance in sample sizes. As the UC sample number is approximately 4 times larger than the CD sample size the variance of UC group is lower, giving higher t-scores in the analysis.

Other gene expression studies of IBD mucosal samples have reported expression differences between UC and CD, and built classifiers based on these results [10], [12], [24]. All of the classifiers are yet to be confirmed in clinical practice. Based on this observation of similarities between UC and CD gene expression, we chose to focus on defining the consensus set of genes and processes describing IBD, as observed when using whole genome gene expression analysis.

#### UC and CD samples share similar expression patterns related to T helper cell subtypes

Disease-specific gene co-expression networks were generated by computing Pearson correlation coefficients for the 4227 and 8376 differentially expressed genes in CD and UC respectively. Network analysis for un-inflamed tissue was not undertaken due to low numbers of differentially expressed genes. For each inflamed network, correlations were measured across normal and disease tissue. The CD-specific network (CD.N) and UC-specific network (UC.N) were partitioned into 13 and 9 modules with at least 30 genes. The largest modules in CD.N and UC.N contained 1261 and 4500 genes respectively. Subsequently, an enrichment analysis of each module identified over-represented Gene ontology (GO) terms. The complete lists of enriched GO processes are provided in Table S3 (for CD.N) and Table S4 (for UC.N). To computationally estimate functional similarities between CD.N and UC.N modules, Jaccard coefficient was calculated for all module pairs identified by the WGCNA. The largest overlap was observed between CD.N module 7 and UC.N module 3 (Jaccard  =  0.14), enriched for “T cell activation”. This similarity between “T cell activation” modules prompted further analysis of gene expression related to T cell differentiation in UC and CD.

The nature of T helper cell development in IBD has been an area of discussion for several years. The predominating theory has been that the adaptive immune response in CD is dominated by Th1 lymphocytes and in UC by Th2 lymphocytes. The identification of Th17 as a unique subset of T helper cells has challenged this view, and today some argue that the adaptive immune response in CD, and to some degree UC, is dominated by Th1/Th17 cells [6]. However, there are still unanswered questions regarding the role of Th2 activation in UC. A set of genes whose expression is known to be related to the four T helper cell lines Th1, Th2, Th17 and Treg were collected. Extracting the differential expression levels given as log2 fold change (log2 FC) and corrected p-values (p) for these genes allows for a characterization of T helper cell function in IBD.

Th1-related transcription factors *STAT1* (log2 FC: 1.62/1.64 for CD/UC, p<0.001) and *STAT4* (log2 FC: 0.55/0.76, p<0.005), as well as the classical Th1 cytokine *IFNG* (log2 FC:0.76/1.09, p<0.001) show increased expression in both CD and UC, a pattern expected during an active Th1 response. This is further supported by the increased expression of *CXCR3* (log2 FC: 0.54/0.42, p 0.001/<0.001), a Th1-associated chemokine receptor[33]. Interestingly there was a small increase in expression of the Th2 transcription factor *GATA3* in UC (log2 FC: 0.11, p: 0.01), but no increase in *IL4* or *STAT6* expression, suggesting an IL4/STAT6-independent activation of the Th2-related transcription factor GATA3. Jenner et al. has suggested an explanation for this unsupported expression of GATA3 by demonstrating that GATA3 may also be expressed in Th1 cells[34]. In this study GATA3 was shown to bind to the promoters of Th1-related genes in a pattern similar to that of the Th1 transcription factor TBX2, suggesting that the absence or presence of TBX2 determines cell lineage [34], [35].

Th17-associated genes are up-regulated in both CD and UC mucosa. The effector cytokines *IL17F* (log2 FC: 0.37/0.24, p<0.002), *IL26* (log2 FC: 0.23/0.24, p: 0.03/<0.001) and *TNF* (log2 FC: 0.49/0.73, p: 0.01/<0.0001) were all up-regulated in CD and UC, suggesting the presence of active Th17-cells in the inflamed mucosa of IBD patients. This is further supported by the overexpression of Th17-associated transcription factors *STAT3* (log2 FC: 0.57/0.58, p<0.001) and *RORA* (log2 FC 0.54/0.48, p: (0.002/<0.001). The cytokines *IL1A* (log2 FC: 1.01/1.45, p<0.001), *IL1B* (log2 FC: 2.38/3.07, p<0.001) and *IL6* (log2 FC: 0.65/1.14, p: 0.01/<0.001), known to stimulate Th17 differentiation, were also up-regulated in IBD tissue[36]. Both Th17-associated chemokine-receptors *CCR4* (log2 FC: 0.26/0.24, p 0.008/<0.001) and *CCR6* (log2 FC: 1.01/0.93, p<0.001), and the Th-17 associated receptor KLRB1/CD161 (log2 FC: 0.83/0.73, p 0.02/<0.01) are differentially expressed in both CD and UC, further substantiating the validity of this observation[37], [38].

There is little classical Treg-related expression, with only *STAT5A* exhibiting a marginal up-regulation in CD and UC (log2 FC: 0.35/0.50, p <0.05). However, both subunits of the Treg-cytokine IL35, *IL12A* (log2 FC: 0.17/0.24, p 0.02/0.002) and *EBV3* (log2 FC: 0.95/0.81, p <0.001), were over-expressed, while their other potential co-factors, *IL12B* and *IL27A*, had no significant increase in expression. IL35 is a newly discovered cytokine produced by T regulatory cells [39], [40]. A Spearman correlation analysis of the subunits corresponding to IL27 (*IL27A* and *EBI3*), IL35 (*EBI3* and *IL12A*), IL23 (*IL23A* and *IL12B*) and IL12 (IL12A and IL12B) had the following rho values; IL27:0.04, IL23:0.003, IL12: 0.13 and IL35: 0.41. This could suggest an IL35-mediated Treg activity in the inflamed mucosa of IBD patients.

An interesting distinction between UC and CD expression was seen in *IL23A*-expression, with a significant log2 FC of 0.92 in UC but no significant difference in CD. The other subunit of IL23, *IL12B*, exhibited no DE in either UC or CD. However, one cannot exclude differential expression of *IL12B*, as expression levels are below the detection limits in the microarray analysis.

Overall, the interpretation of the data in this setting confirms differentiation of activated T cells to Th1 and Th17 cells in both UC and CD, with an apparent absence of Th2-related differential expression. The expression profile of the selected genes in UC and CD are similar in this study, suggesting similar T helper cell population in the mucosa, at least under chronically active conditions.

#### Antimicrobial peptide genes are overexpressed in both UC and CD

There is increased expression of many AMPs in both inflamed UC and CD mucosa, with only a few interesting exceptions. Defensin 1 beta (*DEFB1*) is significantly down-regulated in both UC (log2 FC -2.19) and CD (log2 FC -1.89), while the gene encoding liver expressed antimicrobial protein 2 (*LEAP2*) showed a similar, but less prominent regulation (UC: log2 FC -0.78, CD: log2 FC -0.68). The loss of DEFB1 expression has previously been reported by Arijs et al., who suggested that alterations may be due to a loss of epithelial tissue in inflamed colon [21]. Our results suggest an alternative explanation. A work by Peyrin-Biroulet et al. describes the role of DEFB1 in maintaining homeostasis, and explores the regulation of colonic DEFB1 expression[41] The study demonstrates that DEFB1 expression is under transcriptional control of PPARG. There was a significant down-regulation of peroxisome-proliferator receptor gamma (*PPARG*) in both UC (log2 FC -1.35) and CD (-1.14), and a strong correlation (rho 0.73) between expression of *PPARG* and *DEFB1* suggesting that *DEFB1* down-regulation could be a result of loss of *PPARG* expression. However, further exploration is needed to conclude with a causal effect of the observed correlation.

### Meta-analysis - NTNU data and publicly available datasets

#### Meta-analysis shows intra-experiment differences in power, but good correlation in regulation

Data from all available datasets fulfilling the search criteria defined in the methods section were included in a meta-analysis to identify gene regulation and processes that were stably correlated with disease status over many analysis settings. The rationale was that regulation consistently identified over a cross-platform, cross-laboratory meta-analysis could be seen as externally verified, thereby increasing the validity of conclusions. Details of the datasets are provided in Table 2. The comparisons were performed both on the gene expression and GO category levels. For the gene level analysis, comparisons were made between both t-score (Figure S1) and by ranking of genes (Figure S2). The rank-based analysis was used to identify an appropriate number of genes to use in the t-score analysis. The number of genes used was chosen based on the top union score of the rankings, as calculated using GeneSelector[42]. The gene comparison demonstrated a wide distribution in the statistical power of the tests performed, with a spread in differentially expressed genes from 7500 to 30 in the comparison UC vs. N. All sets containing both UC and CD samples exhibited a higher number of DE genes for the UC vs. N test than CD vs. N test. However, the cluster analysis does not identify a separation between UC and CD on the basis of mucosal gene expression. For tests of un-inflamed tissue vs. normal there was little consensus, suggesting that each of these tests capture different variation. For comparisons of tests of inflamed tissue vs. normal there was a good agreement between the sets with regard to t-scores, the closest clustering involving inflamed tissue.

**Table 2:** Meta-analysis data source.

Name	Study title	PMID [Ref]	GEO/ArrayExpress accession numbers	N	CD	UC	CDU	UCU	Platform
Ahr	**Intestinal macrophage/epithelial cell-derived CCL11/eotaxin-1 mediates eosinophil recruitment and function in pediatric ulcerative colitis.**	18981162 [21]	GSE10191	11	0	8	0	0	Affymetrix HG-U133 Plus 2.0
Ari	Mucosal gene expression of antimicrobial peptides in inflammatory bowel disease before and after first infliximab treatment	19956723 [20]	GSE16879	6	19	24	0	0	Affymetrix HG-U133 Plus 2.0
Bje	Genome-wide gene expression analysis of mucosal colonic biopsies and isolated colonocytes suggests a continuous inflammatory state in the lamina propria of patients with quiescent ulcerative colitis	19834973 [22]	GSE13367	10	0	8	0	9	Affymetrix HG-U133 Plus 2.0
Car	Activation of an IL-6:STAT3-dependent transcriptome in pediatric-onset inflammatory bowel disease.	18069684 [17]	GSE9686	8	10	5	0	0	Affymetrix HG-U133 Plus 2.0
Gal	Diagnostic mRNA expression patterns of inflamed, benign, and malignant colorectal biopsy specimen and their correlation with peripheral blood results	18843029 [23]	GSE10714	3	8	3	0	0	Affymetrix HG-U133 Plus 2.0
Gyo	Inflammation, adenoma and cancer: objective classification of colon biopsy specimens with gene expression signature	18776587 [16]	GSE4183	8	6	9	0	0	Affymetrix HG-U133 Plus 2.0
Kug	Loci on 20q13 and 21q22 are associated with pediatric-onset inflammatory bowel disease	18758464 [24]	GSE10616	11	32	10	0	0	Affymetrix HG-U133 Plus 2.0
Nob	Regional variation in gene expression in the healthy colon is dysregulated in ulcerative colitis	18523026 [13]	GSE11223	63	0	62	0	61	Agilent Whole Human Genome microarray
Ols	Diagnosis of ulcerative colitis before onset of inflammation by multivariate modeling of genome-wide gene expression data	19177426 [11]	GSE9452 (UC samples) and GSE11831 (controls), E-TABM-118 (CD samples)	17	0	8	0	9	Affymetrix HG-U133 Plus 2.0
vbG	NTNU study		E-MTAB-184	20	8	37	19	44	Illumina human HT-12 expression BeadChips
Wu	Genome-wide gene expression differences in Crohn's disease and ulcerative colitis from endoscopic pinch biopsies: insights into distinctive pathogenesis.	17262812 [10]	GSE6731	4	7	5	12	4	Affymetrix HG -U95Av2

**Figure 2:**
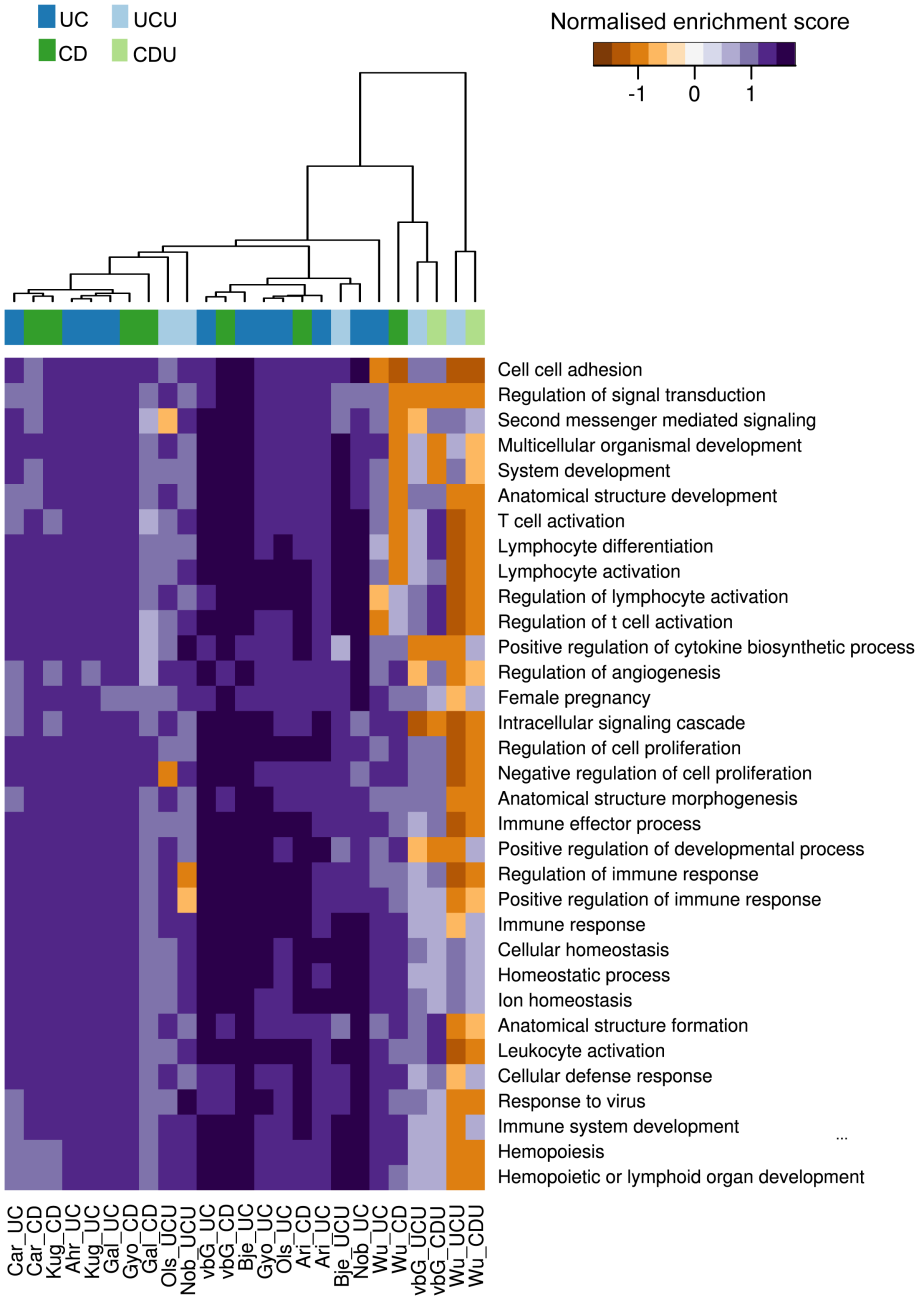
GSEA analysis of all data sets. The figure shows a heat map of GSEA scores for the GO categories selected in the rank-based analysis. Each column in the figure represents the result for one comparison against normal control, with sample source and test group given as column name. The connection between each columns source abbreviation and its related dataset(s) and article(s) are given in table 2.

Information on all data sets used in meta-analysis. Column “Name” refers to abbreviations used in Figure 2- and Figure S1–S5. “Study title” refers to name of original source article, with reference given in column “PMID [Ref]”. Column “No. samples” gives number of samples in each group in the relevant data set, with the following group names: N – Normal controls, CD – Crohn’s disease, UC – Ulcerative colitis, CDU – Un-inflamed Crohn’s disease and UCU – Un-inflamed ulcerative colitis. Column “Platform” refers to microarray technology used in the relevant analysis.

There was a high “intra-experiment” similarity, with lists from the same study clustering as neighbours independently of diagnosis. Strong tests of inflamed vs. un-inflamed samples with many DE genes clustered together. The strength of the sets appeared to be the product of two parameters; many samples or well controlled sample groups. Ahrens et al. have relatively few samples (13 patients) in their UC group, but many DE genes in the UC vs. N comparison (>7500) were identified. The samples used in this comparison were uniquely controlled, with a focus on un-medicated paediatric patients. In the dataset collected by Arijs et al. all samples had high degree of inflammation, taken from patients’ refractory to corticosteroids/immunosuppressant[21]. In our dataset the number of inflamed UC samples was 37, a number of samples that was sufficient to compensate for the relative heterogeneity in sample set, and resulted in a set of approx. 7500 DE genes.

Some comparisons were divergent. The UCU vs. N test based on samples from Olsen et al. cluster with the affected group, showing high t-scores for genes typically DE in inflamed tissue. This corresponded well with conclusions in the article describing the dataset, suggesting a pre-inflammatory state in un-inflamed tissue from patients diagnosed with UC[12]. The expression pattern seen in this set of sample was however not reproduced in the other sets of unaffected samples.

#### Gene set analysis of imported data sets identifies consensus processes important in IBD pathology

By using gene set enrichment analysis the results from all the data sets could be compared at a more general level, potentially identifying the fundamental processes underpinning IBD. This reduction into GO categories also helped overcome difficulties in comparing results from many analyses, as similarities in a gene category could be found even when methodological differences limited any gene-for-gene comparison [43]. An initial analysis was undertaken to identify the appropriate number of GO categories to use in the comparison. The result from this analysis is provided in Figure S3. Figure 2 is a summary of the finds, where categories were chosen on the basis of all data as outlined in the methods section. In this figure all GSEA scores were included, regardless of q value. A supplementary figure with a q value cut-off of 0.25 was included as Figure S4. The analysis showed few gene sets with a significant normalised enrichment score (NES). However, as is seen in figure 2, there is a remarkable consistency in the NES for top ranked categories across studies, suggesting that similar processes are dominating all gene lists based on inflamed samples. As was demonstrated in the t-score based analysis, the cluster analysis readily separated inflamed from un-inflamed comparisons, showing little separation of CD and UC sets. The separation between strong and weak data sets was also repeated.

The list of GO categories was dominated by inflammation-related categories both related to innate and adaptive immunity responses. There were several categories describing cell proliferation, thought to be activated due to the increase in epithelial cell regeneration as a response to tissue damage. Interestingly, several categories related to angiogenesis were broadly activated, supporting the notion that this may be an important process in IBD[44].

#### Both T helper cell and AMP observations are confirmed by meta-analysis

**Figure 3:**
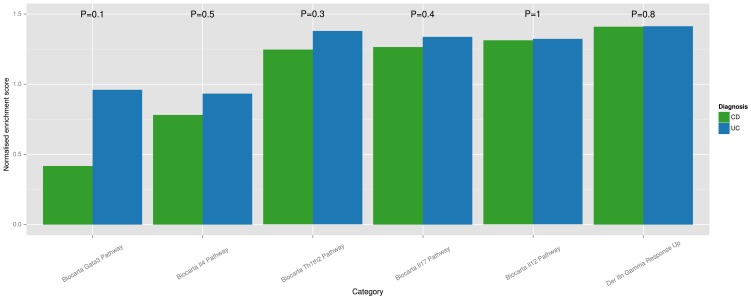
T helper cell associated GSEA analysis. A subset of gene categories thought to represent T helper cell differentiation was selected from MSigDB. The bar plot is a summary of results from GSEA analysis of this subset, where each bar represents the mean GSEA score for a sample group (UC or CD) for the GSEA category in question. A Wilcox rank-sum test was performed between disease groups fails to identify a difference between GSEA scores for the selected categories.

GSEA analysis based solely on categories related to T helper cell differentiation was performed as described in the methods section. A plot of mean NES scores for UC and CD is given in Figure 3. A Wilcox rank-sum test between UC and CD for each gene category demonstrated no significant difference in NES score for UC and CD. The analysis demonstrated the highest mean NES score for Th1 and Th17-related categories, while the Th2-related categories showed the lowest mean GSEA score for both UC and CD. The GATA3 category showed the largest difference in activity when comparing UC and CD. Of all the categories, this also has the smallest p-value (0.1).

**Figure 4:**
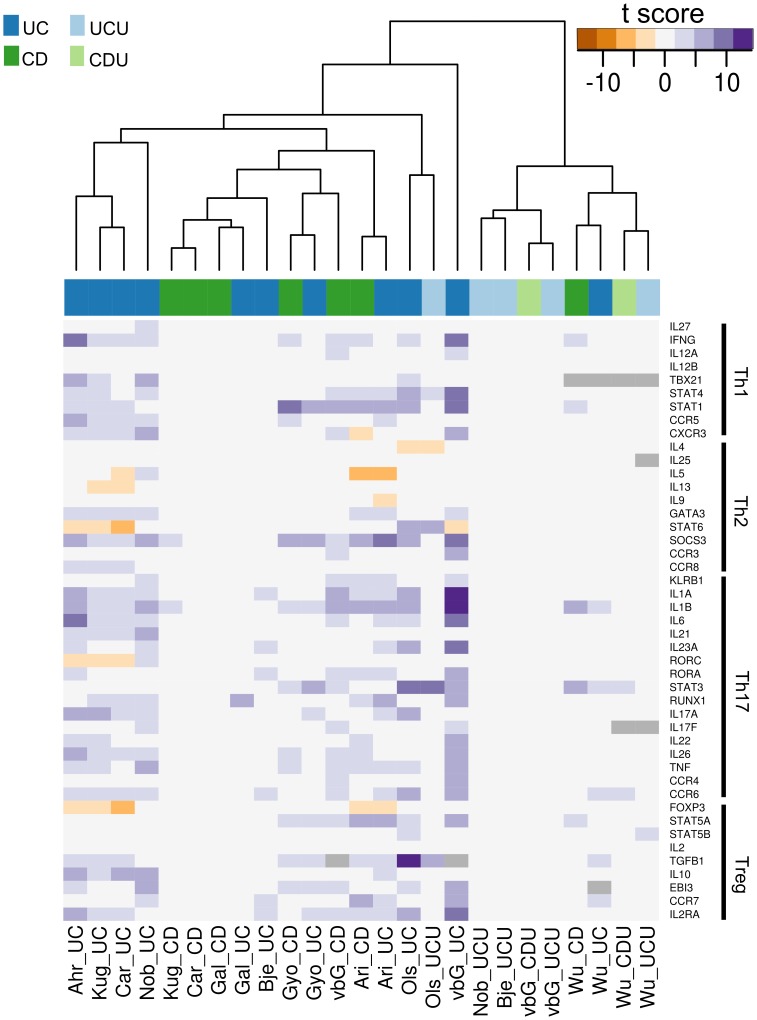
T helper cell-associated genes. The figure shows a heat map of t-scores (corrected p-val <0.05) for genes related to T helper cell differentiation and function. Figure S4 shows the same data with a no p-value cut-off. Genes are grouped in the Th sub-categories Th1, Th2, Th17 and Treg. Each column in the figure represents the result for one comparison against normal control, with sample source and test group given as column name. The connection between each column’s source abbreviation and its related dataset(s) and article(s) are given in table 2. Some sets lack measurements for certain genes, in which case a grey marking is used.

**Figure 5:**
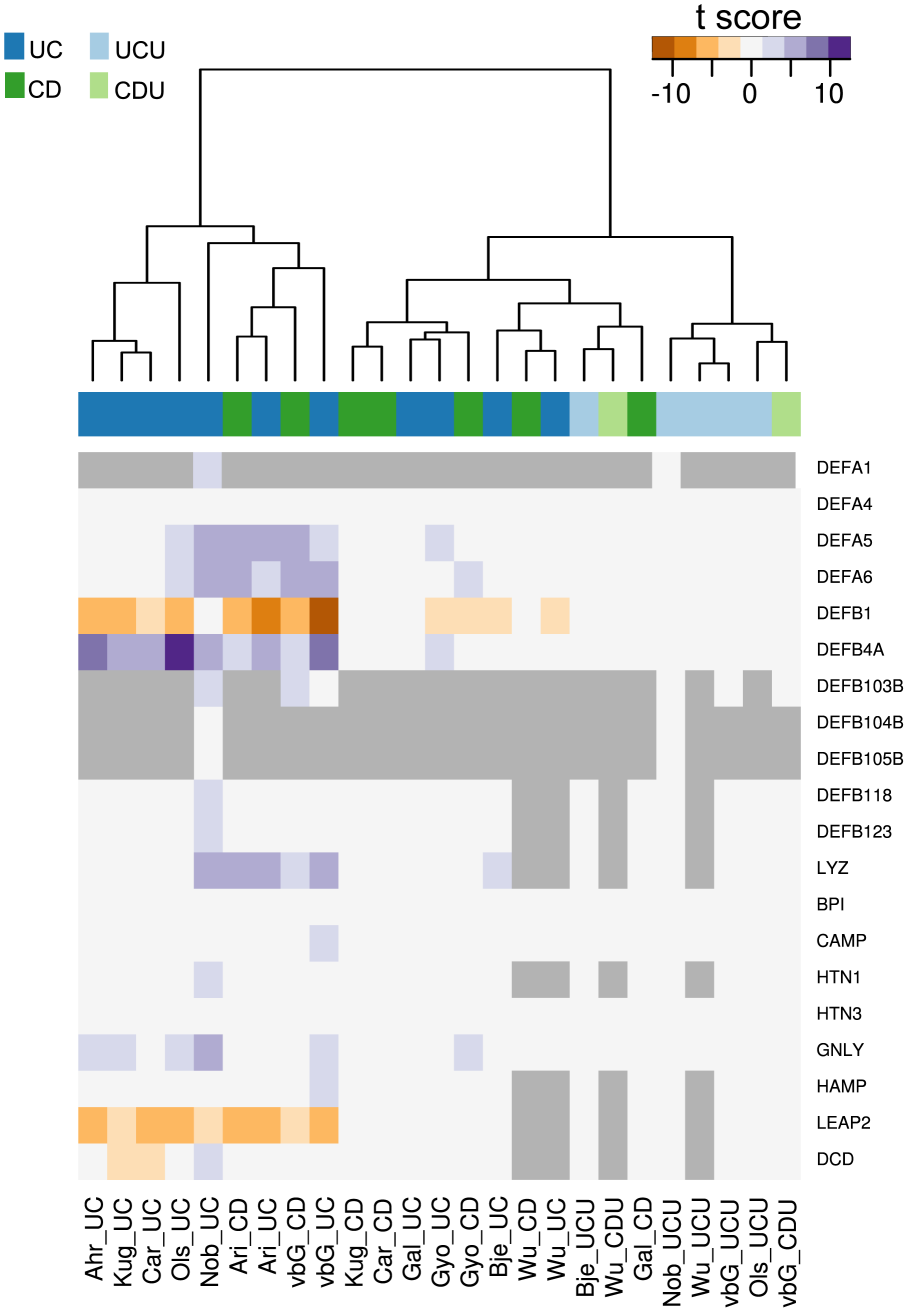
Antimicrobial peptide gene expression. The figure shows a heat map of t-scores (corrected p-val <0.05) for genes coding for known antimicrobial peptides. Figure S5 shows the same data with no p-value cut-off. Each column in the figure represents the result for one comparison against normal control, with the sample source and test group given as column name. The connection between each column’s source abbreviation and its related dataset(s) and article(s) are given in table 2. Some sets lack measurements for certain genes, in which case a grey marking is used.

Two lists of gene names were assembled to further investigate T helper cell differentiation and AMP expression in inflamed colonic IBD mucosa. The list of AMPs was based on work by Arijs et al[21]. here was a broad consensus between gene regulation observed in the presented dataset and the meta-analysis both in the subset of T helper cell-associated and AMP genes. Two heat maps were constructed from each gene set, one using only significant t-scores and one with all t-scores irrespective of significance, the rationale being that even non-significant regulation could help interpretation when supported by significant regulation in other analyses. Figures based on scores with corrected p-value <0.05 are included as Figures 4 and 5. The figures based on all scores are given as Figure S5 and S6. Applying p-value cut-off reveals the lack of statistical significance of observed gene regulation for many of the data sets.

The observations made based on our dataset were broadly confirmed by all imported datasets, even when t-scores were not significant. There were few differences in t-scores between UC and CD. *IL23A* expression was only identified as significantly increased in UC vs. N tests. This was an interesting finding when viewed in the light of previous research exploring the IL23-axis in IBD [45], [46]. Animal models have suggested an important role for IL23 in Th17 proliferation, and an IBD-susceptibility SNP has been identified in the IL23 receptor [46], [47]. The different *IL23A* levels detected in inflamed tissue from UC and CD patients suggest distinct roles for this cytokine in the two diseases. Our dataset is the only analysis showing a DE for *IL6* in CD vs. N, with all other DE shown in UC vs. N tests. Our set together with the data of Noble et al. also identified a DE of *IL17F*, while the other data sets found *IL17A* as DE. A possible explanation for this might be differences in technology, as *IL17F* was found DE on Affymetrix chips, while *IL17A* was found on Agilent and Illumina microarrays.

An interesting observation was the down-regulation of *RORC*. RORC is seen as a key transcription factor for Th17 cells, that together with STAT3 and RUNX1 promotes the transcription of the effector cytokines IL17A/F, IL21, IL22, IL26 and CCL20[48]. This down-regulation was unexpected, as both key regulators of *RORC* expression (*TGFB* and *STAT3*), the transcription co-factors (*RUNX1* and *STAT3*) and the effector cytokines were all over-expressed in many of the datasets included in the analysis. However, the closely related paralog to *RORC*, *RORA*, was over-expressed in several datasets. Studies in mice have demonstrated that RORA expression is sufficient to promote Th17 differentiation, possibly explaining the regulation seen in this meta-analysis[49]. It must also be stressed that even though there was a down-regulation of *RORC* expression, there was no evidence of loss of *RORC* function, possibly suggesting that the relative down-regulation is not enough for RORC to be a limiting factor in the combined transcriptional regulation of RORC, RORA, RUNX1 and STAT3. Interestingly, the sets showing the most distinct regulation of *RORC* was based mainly on un-medicated paediatric patients (Ahrens et al. and Carey et al.), together with data from Kugathasan et al. where no patient age information was given. The same datasets also showed no significant regulation of *STAT3*.

Almost all datasets exhibited over-expression of suppressor of cytokine signalling 3 (*SOCS3*), a gene closely associated with Th2 cell development, while there was a broad down-regulation of other Th2-associated transcription. This apparent paradox has been observed in CD before[50]. It might arise due to another role of SOCS3, namely as an inhibitor of naïve CD4+ cell differentiation to activated T cells. SOCS3 inhibits the production of IL-2, a cytokine crucial for the activation of T cells, effectively inhibiting initial T cell activation. The over-expression of *SOCS3* in this setting might therefore be the result of an effort to limit the activation of T helper cells in the inflamed mucosa, while the increased expression of *STAT3* might be the result of SOCS3-independent activation via IL10[51], [52]. It is also possible that the *SOCS3* expression detected stems from cells other than the lymphocytes, as both neutrophils, macrophages and epithelial cells have been shown to express *SOCS3* in IBD mucosa[53].

In the AMP set the pattern of expression seen in our dataset was reproduced in the imported sets as shown in Figure 5. The two down-regulated AMPs *DEFB1* and *LEAP2* exhibited similar down-regulation in the other sets, while at the same time confirming the observed up-regulation of AMPs in inflamed mucosa. The down-regulation of *PPARG* was also broadly confirmed in the imported data (data not shown).

## Conclusion

The present study is the to-date largest microarray gene expression study examining both inflamed and un-inflamed samples from colonic mucosa of IBD patients. Uniquely, the analysis also includes a gene co-expression network analysis and a meta-analysis of comparable IBD datasets. The analysis shows a similarity between the gene expressions in inflamed mucosa from UC and CD patients, which is confirmed by hierarchical clustering of several independent data sets. This suggests that once established the inflammatory mechanisms at mucosal level are largely the same for the two diseases. Our analysis further demonstrates that the T helper cell-related expression found in the inflamed colonic mucosa of all IBD patients is dominated by Th1/Th17-related expression, with little to no signs of Th2 differentiation. There was no significant difference between GSEA scores for T helper cell related categories between UC and CD. The mean GSEA score was highest for categories related to Th1/Th17, and lowest for Th2-related categories. The only selected gene showing a clear difference in expression between UC and CD was *IL23A*, consistently showing DE in UC vs. N comparisons. This distinction between UC and CD T cell expression seems to be highly reproducible, and is of particular interest given previous research emphasising the importance of the IL23-axis in IBD. Another feature very similar across the diagnoses and the different datasets is the significant change in expression of antimicrobial peptides, the AMPs. The expression of many AMPs in inflamed IBD mucosa is generally increased when compared to un-inflamed mucosa, with the exception of *DEFB1* and *LEAP2*. In the in-house data, the loss of *DEFB1* is highly correlated with loss of *PPARG* expression, a gene known to exert a promoting effect on DEFB1. In addition, mucosal biopsies from un-inflamed colon in patients with IBD had gene expression patterns almost identical to healthy control subjects, with only CDU displaying a few differentially expressed genes in the NTNU dataset.

This IBD WGGE study for the first time integrates external data sets, providing a sound background for further research on IBD pathobiology. The chosen strategy of focusing on consensus genes and processes over several datasets facilitates result interpretation and validation.

## Materials and Methods

### Sample material

Samples were collected from an IBD cohort at St. Olav’s University Hospital, Trondheim, Norway. Study participants were patients admitted to the Department of Gastroenterology for colonoscopy. Participants were either diagnosed with UC or CD, or were admitted for diagnostic colonoscopy due to symptoms unrelated to IBD. Patients were only included as normal controls after all clinically indicated examinations had concluded no signs of gastrointestinal disease. In the IBD groups (UC and CD), four endoscopic pinch biopsies were taken from macroscopically maximally inflamed mucosa, as well from the hepatic flexure in cases where this was found to be macroscopically un-inflamed UC unaffected (UCU) and CD unaffected (CDU)). Two UCU samples, 228 F and 118 F were obtained from ascending colon and rectum, respectively. For the normal (N) group, four biopsies were taken from the hepatic flexure. One biopsy from each area was fixed in 4% buffered formaldehyde, while the three remaining biopsies were snap frozen and stored in liquid nitrogen.

### Ethics statement

Written informed consent was obtained from all participants, and the study was approved by the Regional Medical Research Ethics Committee (approval no 5.2007.910). The study was registered in the Clinical Trials Protocol Registration System (identifier NCT00516776).

### Microarray analysis

#### RNA extraction and quality control

Frozen biopsies were homogenized in lysis buffer using a rotating knife homogenizer (Zanke & Kunkel IKA-Laboratorie Technik, Staufen, Germany). Total RNA was extracted using the Ambion mirVana miRNA Isolation Kit (Applied Biosystems, CA, USA). Quality control was performed for each extract. Quantity and purity of RNA was assessed using a NanoDrop Spectrophotometer (Thermo Scientific, DE, USA), and the integrity of isolated RNA was determined using Bioanalyzer (Agilent Technologies, CA, USA). Samples were only included in subsequent analysis if the RIN value > 7. Isolated RNA was stored in cryo-tubes in liquid nitrogen.

#### Hybridization

250 ng total RNA from each sample was used to generate biotinylated, amplified cRNA using the Illumina TotalPrep RNA Amplification kit (Applied Biosystems/ Ambion, Austin, TX, USA). cRNA was stored at −80°C, and the concentration of each cRNA sample was adjusted to 150 ng/ µl prior to hybridization. The samples were all hybridized in parallel on Illumina human HT-12 expression BeadChips (Illumina, San Diego, CA, USA) and scanned on an Illumina BeadStation. Results from the microarray analysis are available at ArrayExpress E-MTAB-184.

### Differential expression analysis

Expression data analysis was performed using the R software environment for statistical computing[54]. Methods supplied in the Bioconductor package were used for import, quality assessment, normalization, filtering and statistical analysis of the expression data [31]. Data was log-transformed and quantile normalized. Probes not corresponding to an ENTREZ ID were removed. In cases where several probes corresponded to one ENTREZ ID, the probe showing the highest variance over all samples was chosen for further analysis. Initial interpretation of data was done using the unsupervised method of Principal Component Analysis (PCA). Final differential expression analysis was performed using LIMMA linear models with least squares regression and empirical Bayes moderated t-statistics[55]. P-values were adjusted for multiple comparisons using the Benjamini Hochberg false discovery rate correction (FDR). A corrected p-value of 0.05 was chosen as significance level.

### Network analysis

#### Weighted correlation network formation

From the normalized microarray dataset we followed the protocol for Weighted Correlation Network Analysis (WGCNA) [56], [57] to create CD- and UC- specific networks. For each network, only DE genes (Bayes moderated p-values <0.05) in diseased compared to normal samples were analysed and co-expressions were measured across normal and diseased tissue.

#### Gene module detection, comparison, and enrichment

Genes were hierarchically clustered using 1 – Topological Overlap (TO) [58] as the distance measure and modules were determined by applying a dynamic tree-cutting algorithm [56]. TO is a biologically meaningful measurement that reflects the similarity of gene co-expression relationships in the network, while the dynamic tree-cutting algorithm allows identification of clusters in a dendrogram depending on their shape. Similarity between CD and UC disease modules was expressed using the Jaccard coefficient - the ratio of the number of genes common to two modules to the number of total genes in both modules. All modules were enriched for over-represented Gene Ontology (GO) functional terms using the Fisher exact test. As background we used all of the supplied genes that were present in at least one term in the GO.

### Meta-analysis of available data sets

#### Choosing data sets for meta-analysis

The microarray data repositories Array Express and NCBI GEO were searched for datasets containing references to “Inflammatory bowel disease”, “Ulcerative colitis” or “Crohn’s disease” [59], [60]. These datasets where then evaluated further by including only analyses done on pinch biopsies from human colon. Data from printed arrays (oligo/cDNA microarrays) were excluded. Only sets where at least one of the groups UC, CD, UCU, CDU in addition to control individuals (N) were included. The total number of external datasets included in the analysis was 10. A full list of included data sets is given in Table 2

#### Differentially expressed genes meta-analysis

We used the limma software package (ver. 3.12.1) from Bioconductor to assess the effect of DE of IBD subgroups with respect to normal controls. The individual contrast for each dataset was summarised by t-scores and adjusted p-values in the same manner as the original analysis of our data. All analyses following this were based on the resulting “gene x contrast” matrix, where each contrast from each set was represented with two tables containing t-scores and p-values. We determined an aggregated rank score for each gene by a weighted-mean summary across the ranks of inflamed (UC, CD) t-scores. In this method, the weights received are proportional to the logarithm of the number of genes identified as significant in the limma-model, with a minor moderation to accommodate small values. In cases where only a subset of the datasets (due to differences in array types) have a specific gene present we used imputed values for the missing t-scores based on a k-nearest neighbour imputing scheme as implemented in the impute (ver. 1.30.0) Bioconductor package. The imputed values were subsequently removed prior to results visualization.

The (genes x contrasts) table of ranks was visualised as a heat map with genes ordered after aggregated rank score and contrasts ordered by distance to the aggregated rank score. The distance was calculated using linear decay weighting of the Manhattan distance as implemented in the default settings of the GeneSelector package (ver. 2.6.0) and a unionscore was computed from the same package using only the inflamed contrasts. The unionscore measures the similarity between multiple ordered lists by counting the size of the union for each position in the aggregated rank score and normalising with regards to position. A high unionscore indicates high agreement between contrasts. The maximum unionscore was achieved at position 2973 and thus the first 2973 genes were selected for visualising t-scores. The t-scores were visualized in a heat map where both rows and columns were ordered by hierarchical clustering using Euclidean distance and Ward agglomeration. *Gene ontology GSEA meta-analysis*

A GSEA (Gene Set Enrichment Analysis) score was estimated for each of the individual contrasts. The more recent version weighted with correlation to phenotype was used and gene sets were collected from the GO Biological Process part of MSigDB (ver. 3.1) [61]. This relatively reduced set of categories was chosen to improve interpretation, as the goal was not mainly to identify novel categories but rather to provide a well validated representation of the processes at hand in colonic IBD inflammation. A gene set was accepted if it contained between 25 and 1500 genes. Categories were further filtered on q value, including only categories where at least 3 comparisons had q values < 0.25. This resulted in a list of 233 categories. These categories were included in the union score calculation as described for the gene-wise analysis, this time based on ranked q values identifying 33 categories with the top union score. The results were visualised as in the gene meta-analysis. A second GSEA analysis was performed on a subset of the MSigDB categories related to T helper cell differentiation. These categories were “Biocarta GATA3 pathway”, Biocarta IL12 pathway”, “Biocarta IL17 pathway”, “Biocarta IL4 pathway”, “Biocarta Th1Th2 pathway”, “Biocarta IL4 pathway” and “Der INF gamma response up”. A GSEA score was calculated for each of the contrasts, and mean GSEA score for UC and CD contrast were calculated for each category. A Wilcox rank-sum test was performed between GSEA scores for UC and CD.

## Supporting Information

Figure S1 **T-score analysis of all data sets.** A visualisation of the top scoring genes over all available data sets. Each column represents one comparison, with sample group and source given in the column name. Each vertical line represents a t-score from the corresponding analysis. Grey lines replace missing values, where no measurement of the gene in question was given in the source data. The connection between each columns source abbreviation and its related dataset(s) and article(s) are given in table 2.
(TIF)

Figure S2 **Gene rank analysis of all data sets.** Figure illustrating the method used to choose the number of genes used in t-score based comparison of all data sets. Optimal number of genes was chosen at the maximum unionscore as described in Methods section.
(TIF)

Figure S3 **GO rank heat map.** GO rank analysis: Figure illustrating the method used to find the optimal number of GO categories to use in a comparison of all data sets. The optimal number of GO categories (33) was chosen at maximum unionscore as described in methods section.
(TIF)

Figure S4 **GSEA analysis of all data sets with q value cut-off.** The figure shows a heat map of GSEA scores for the GO categories selected in the rank-based analysis. Each column in the figure represents the result for one comparison against normal control, with sample source and test group given as column name. Scores with q-value > 0.25 are removed. The connection between each columns source abbreviation and its related dataset(s) and article(s) are given in table 2.
(TIF)

Figure S5 **T helper cell associated genes.** Figure shows a heat map of significant t-scores for genes related to T helper cell differentiation and function. Genes are grouped in the Th sub-categories Th1, Th2, Th17 and Treg. Each column in the figure represents the result for one comparison against normal control, with sample source and test group given as the column name. The connection between each column’s source abbreviation and its related dataset(s) and article(s) are given in table 2. Some sets lack measurements for certain genes, in which case a grey marking is used.
(TIF)

Figure S6 **Antimicrobial peptide gene expression.** Figure shows a heat map of significant t-scores for genes coding for known antimicrobial peptides. Each column in the figure represents the result for one comparison against normal control, with sample source and test group given as the column name. The connection between each column’s source abbreviation and its related dataset(s) and article(s) are given in table 2. Some sets lack measurements for certain genes, in which case a grey marking is used.
(TIF)

Table S1 A table listing the detailed sample description for all samples included in the microarray analysis.
(PDF)

Table S2 **The full results from the limma analysis of the NTNU data.** P-values given are benjamini-hochberg corrected for multiple comparisons.
(XLS)

Table S3 **csv – Comma separated values.** Over-represented Gene Ontology terms in the Crohn’s disease-specific gene co-expression network.
(CSV)

Table S4 **csv – Comma separated values.** Over-represented Gene Ontology terms in the Ulcerative colitis-specific gene co-expression network.
(CSV)
