# Supplementary figures and images for: Whole Genome Gene Expression Meta-Analysis of Inflammatory Bowel Disease Colon Mucosa Demonstrates Lack of Major Differences between Crohn's Disease and Ulcerative Colitis
Source: PLoS One. 2013 Feb 13;8(2):e56818. doi: 10.1371/journal.pone.0056818 (PMC3572080; doi:10.1371/journal.pone.0056818)

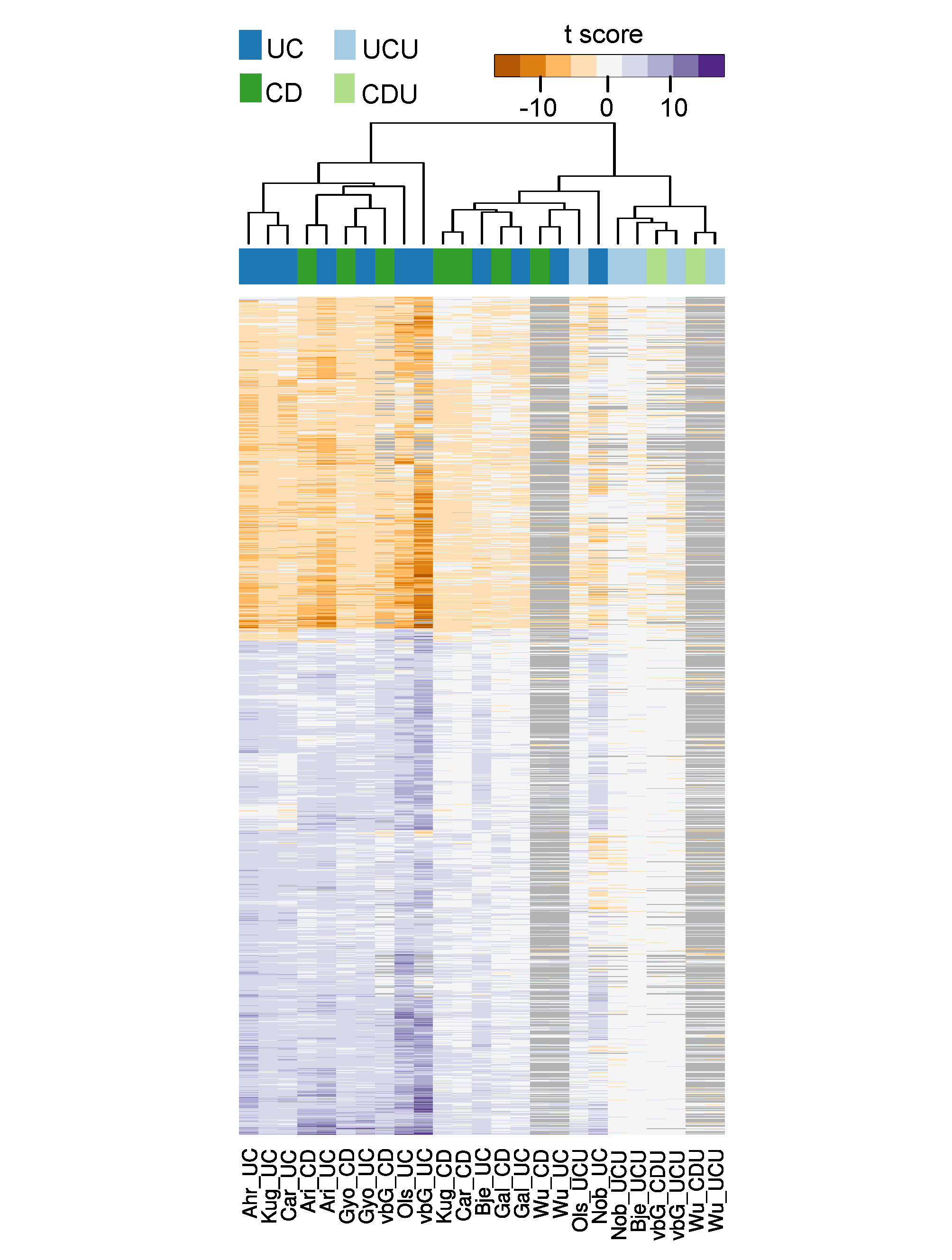

Supplement: Figure S1 — T-score analysis of all data sets. A visualisation of the top scoring genes over all available data sets. Each column represents one comparison, with sample group and source given in the column name. Each vertical line represents a t-score from the corresponding analysis. Grey lines replace missing values, where no measurement of the gene in question was given in the source data. The connection between each columns source abbreviation and its related dataset(s) and article(s) are given in table 2. (TIF) [file pone.0056818.s001.tif]

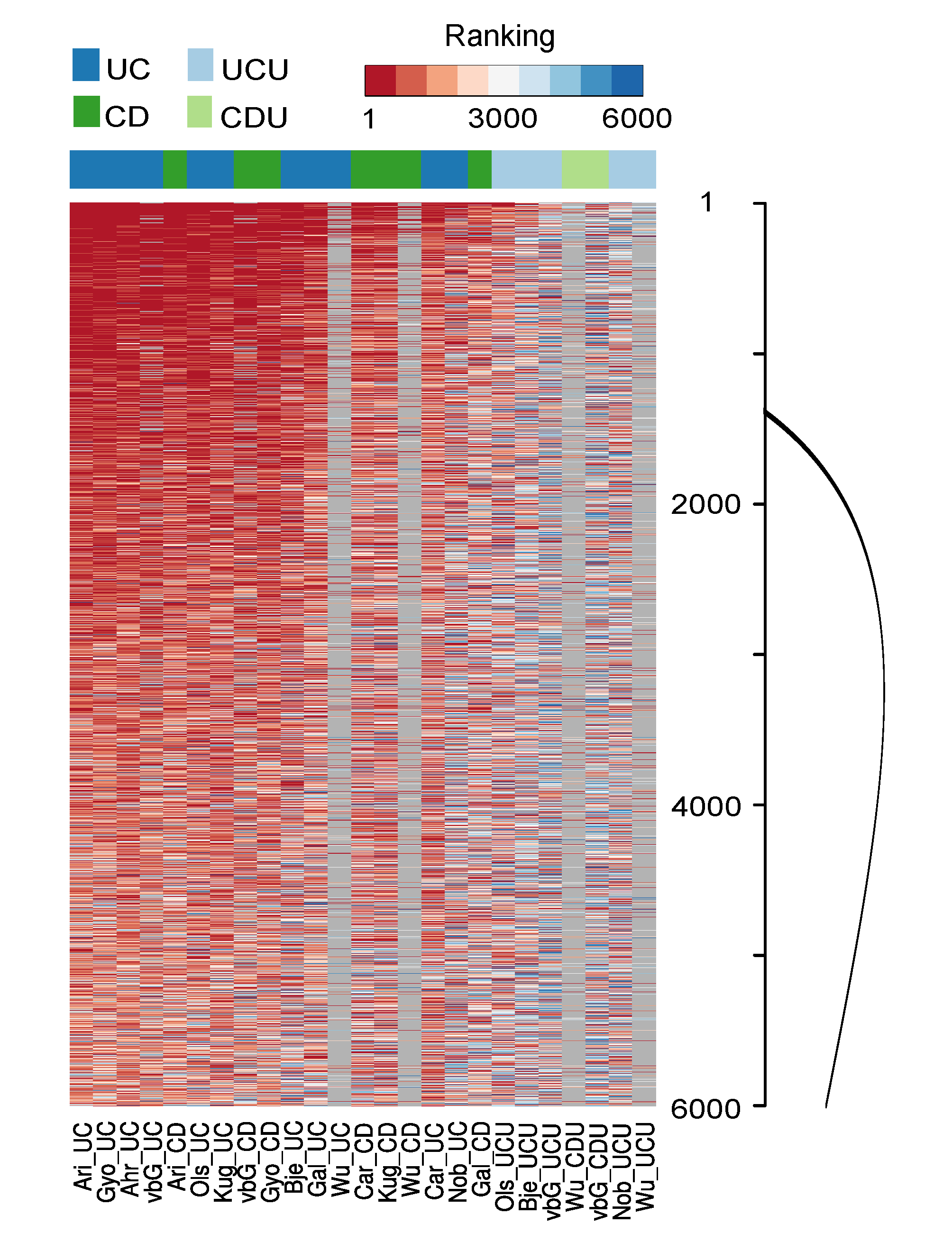

Supplement: Figure S2 — Gene rank analysis of all data sets. Figure illustrating the method used to choose the number of genes used in t-score based comparison of all data sets. Optimal number of genes was chosen at the maximum unionscore as described in Methods section. (TIF) [file pone.0056818.s002.tif]

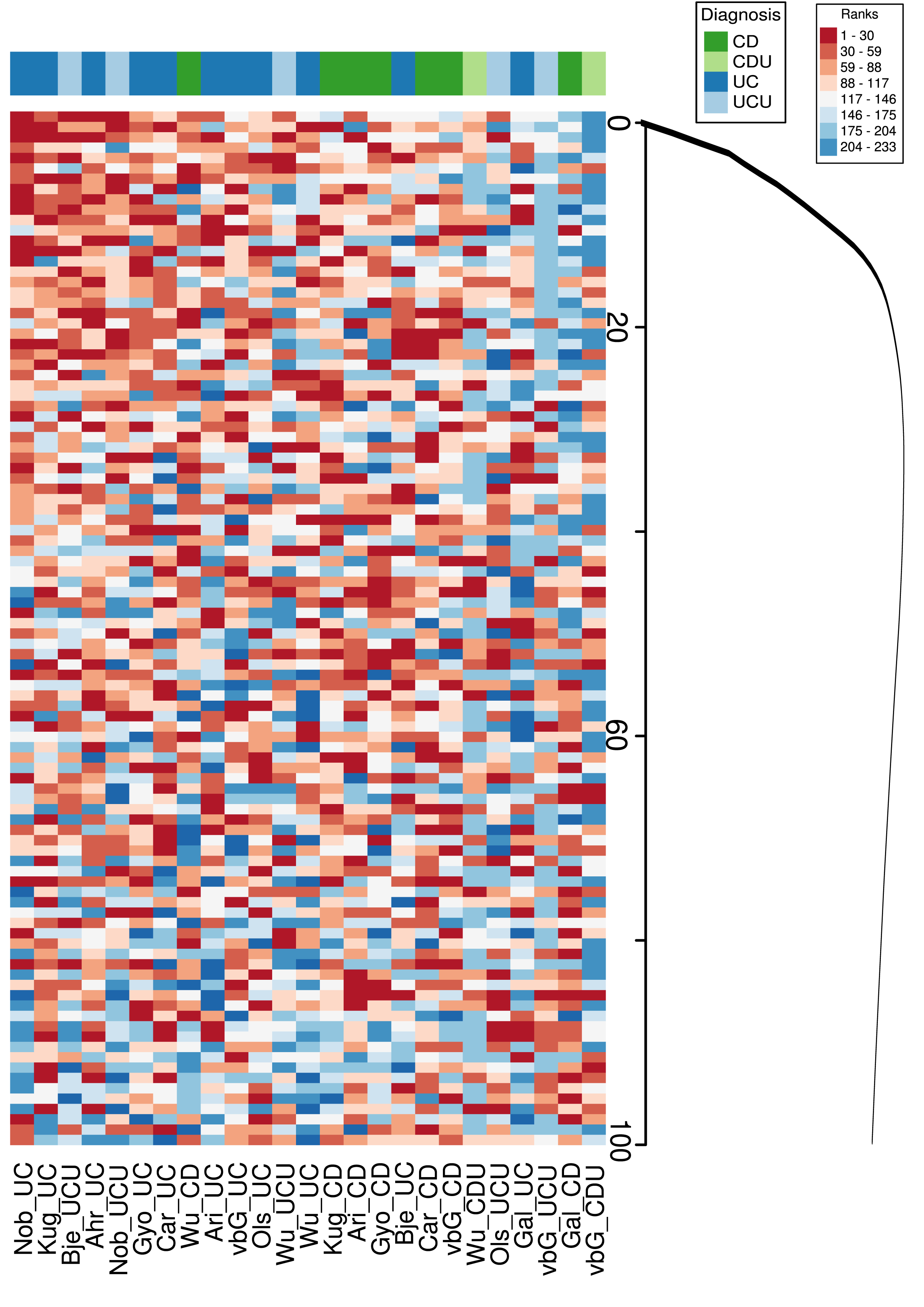

Supplement: Figure S3 — GO rank heat map. GO rank analysis: Figure illustrating the method used to find the optimal number of GO categories to use in a comparison of all data sets. The optimal number of GO categories (33) was chosen at maximum unionscore as described in methods section. (TIF) [file pone.0056818.s003.tif]

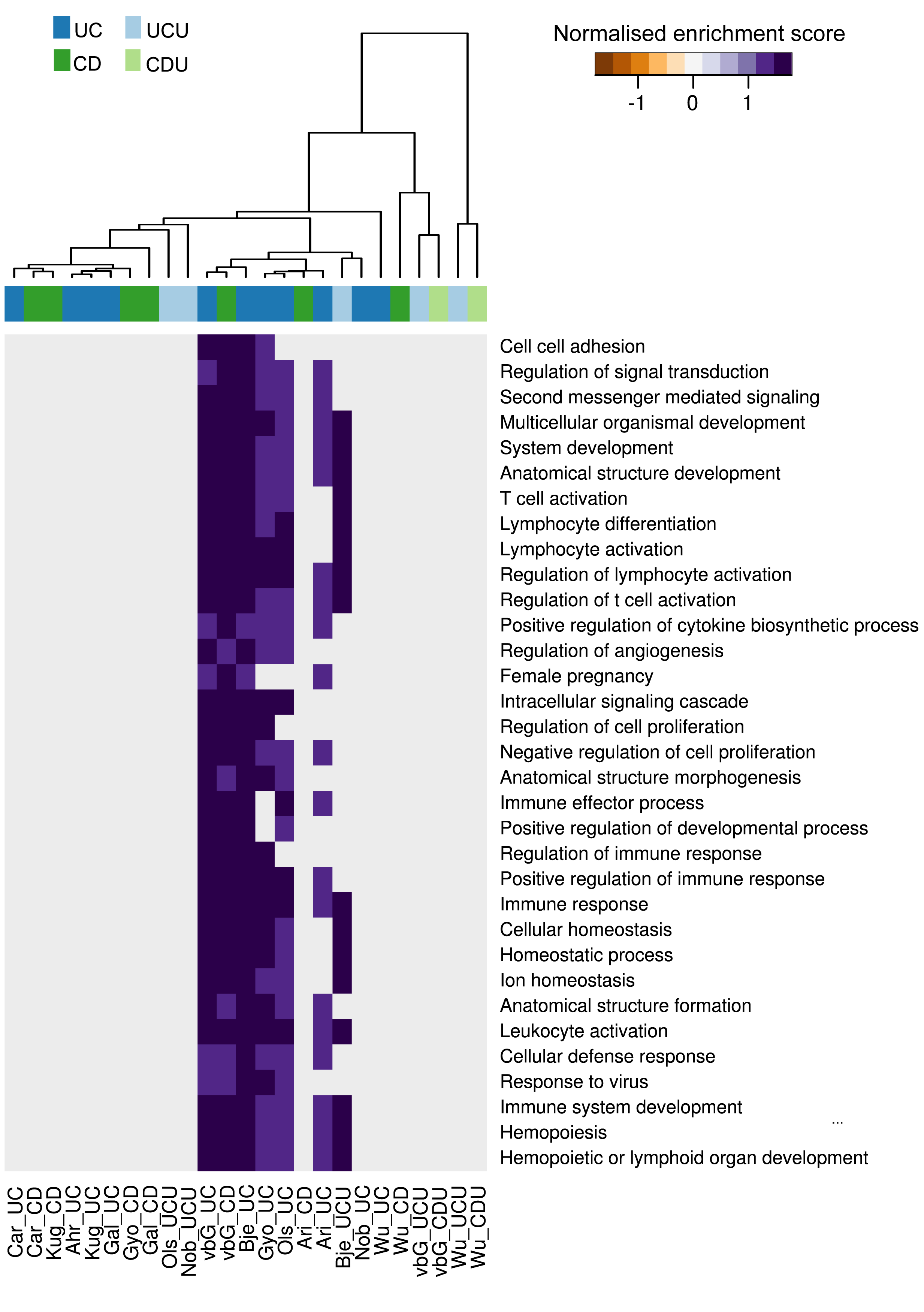

Supplement: Figure S4 — GSEA analysis of all data sets with q value cut-off. The figure shows a heat map of GSEA scores for the GO categories selected in the rank-based analysis. Each column in the figure represents the result for one comparison against normal control, with sample source and test group given as column name. Scores with q-value > 0.25 are removed. The connection between each columns source abbreviation and its related dataset(s) and article(s) are given in table 2. (TIF) [file pone.0056818.s004.tif]

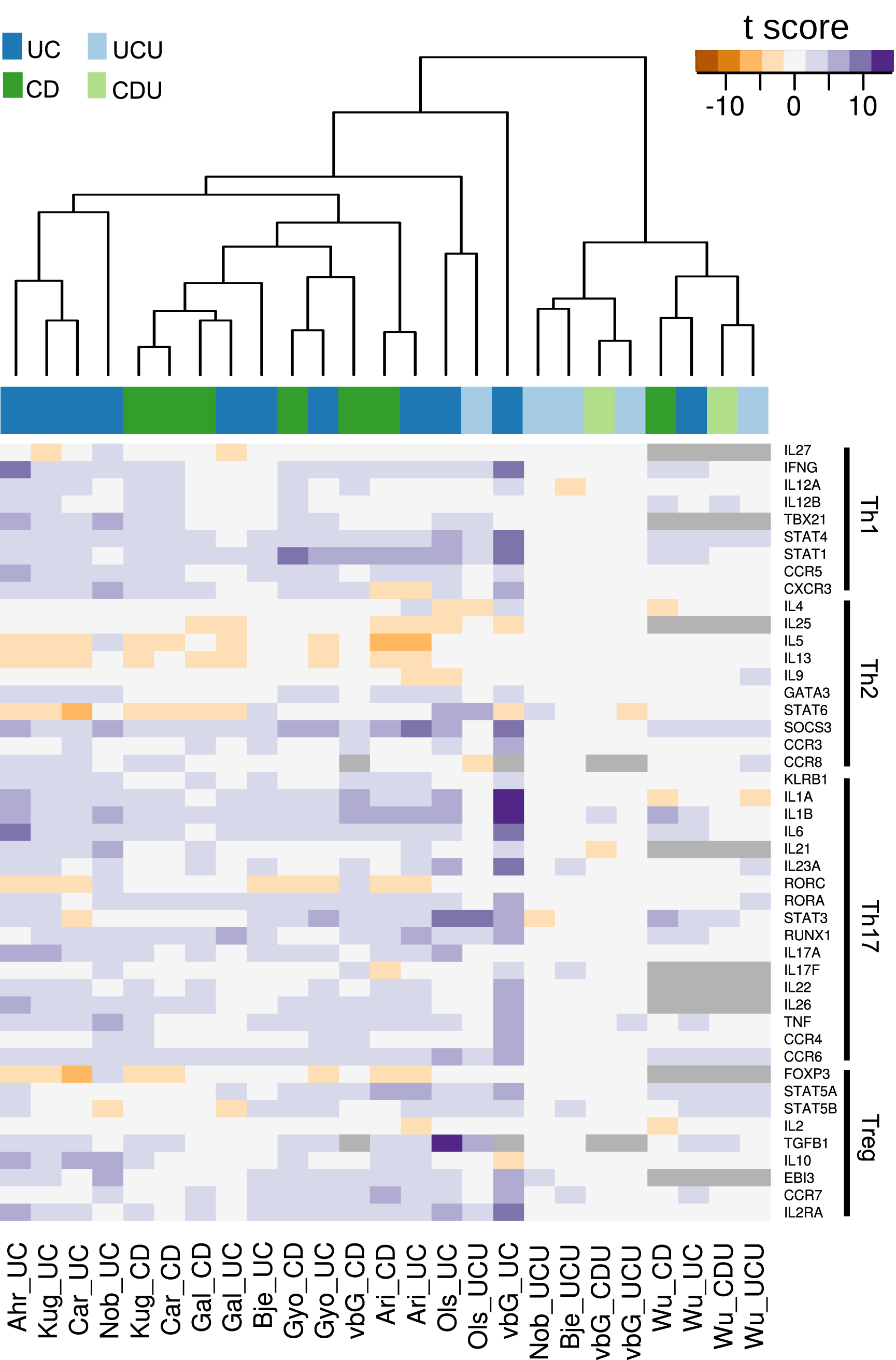

Supplement: Figure S5 — T helper cell associated genes. Figure shows a heat map of significant t-scores for genes related to T helper cell differentiation and function. Genes are grouped in the Th sub-categories Th1, Th2, Th17 and Treg. Each column in the figure represents the result for one comparison against normal control, with sample source and test group given as the column name. The connection between each column’s source abbreviation and its related dataset(s) and article(s) are given in table 2. Some sets lack measurements for certain genes, in which case a grey marking is used. (TIF) [file pone.0056818.s005.tif]

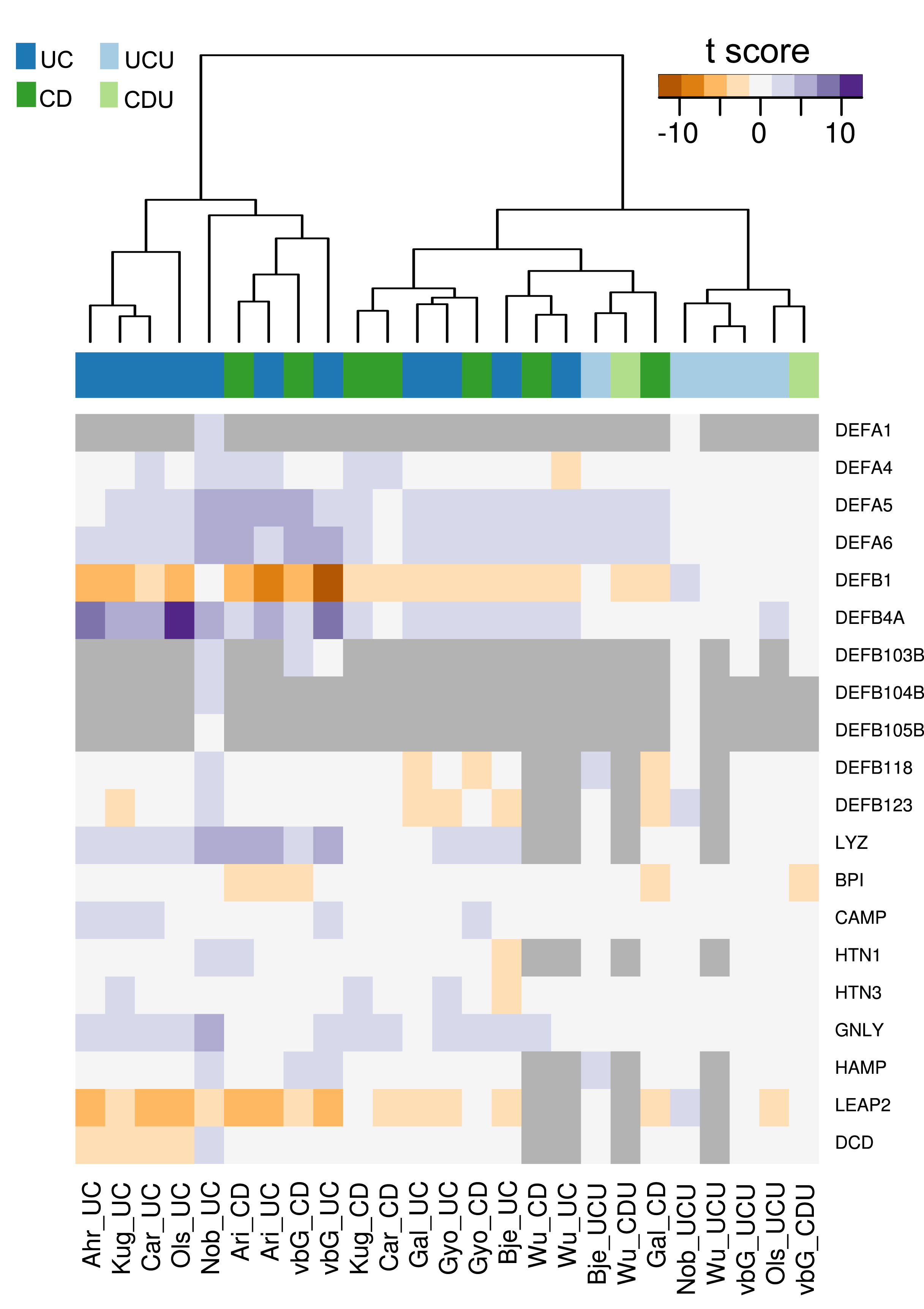

Supplement: Figure S6 — Antimicrobial peptide gene expression. Figure shows a heat map of significant t-scores for genes coding for known antimicrobial peptides. Each column in the figure represents the result for one comparison against normal control, with sample source and test group given as the column name. The connection between each column’s source abbreviation and its related dataset(s) and article(s) are given in table 2. Some sets lack measurements for certain genes, in which case a grey marking is used. (TIF) [file pone.0056818.s006.tif]
