# Supplementary material for: Whole Genome Gene Expression Meta-Analysis of Inflammatory Bowel Disease Colon Mucosa Demonstrates Lack of Major Differences between Crohn's Disease and Ulcerative Colitis
Source: PLoS One. 2013 Feb 13;8(2):e56818. doi: 10.1371/journal.pone.0056818 (PMC3572080; doi:10.1371/journal.pone.0056818)
Supplement: Table S1 — A table listing the detailed sample description for all samples included in the microarray analysis. (PDF) [file pone.0056818.s007.pdf]

Supplementary Table 1: Full sample pool info.

"NA" is given when time of first diagnosis is missing. Sample group abbreviations: UC – Ulcerative colitis, CD – Crohn's disease, N – Normal control. Sex abbreviations: M – Male, F – Female. Sample area abbreviations: R - Rectum, S – Sigmoid colon, D – Descending colon, T – Transverse colon, H – Hepatic flexure, A – Ascending colon, C – Caecum. Medication abbreviations: 5-ASA – 5-aminosalicylic acid, S-ASA – Sulfasalazine. Sample names and info for all samples used in the final analysis of the data given in ArrayExpress E-MTAB-184.

Sample Name	Sample Group	Age (years)	Sex	Sample Location	Time since first diagnosis (years)	Medication
100 F	UC	46	M	R	8	5-ASAS/S-ASA
100 S	UC	46	M	HF	8	5-ASAS/S-ASA
103 F NY	N	46	F	HF	0	
104 F	UC	35	F	HF	25	5-ASAS/S-ASA
104 S	UC	35	F	T	25	5-ASAS/S-ASA
105 F NY	N	71	M	HF	NA	
109 F	N	69	M	HF	0	
110 S	UC	40	M	R	1	
111 S	UC	66	F	S	0.5	
112 F	CD	29	F	HF	10	Corticosteroids
113 F	UC	51	M	HF	7	5-ASAS/S-ASA
115F_4	N	51	M	HF	0	
117 F	UC	38	M	HF	27	
117 S	UC	38	M	R	27	
118 F	UC	29	F	R	6	
119 S	UC	27	F	A	0.5	
119 F	CD	27	F	HF	0.5	Corticosteroids
120 F	CD	20	M	HF	1	Corticosteroids
120 S	CD	20	M	D	1	Corticosteroids
122 S	UC	47	F	S	15	5-ASAS/S-ASA
123 S	UC	36	F	S	5	Corticosteroids
124 S	UC	21	F	D	0.5	5-ASAS/S-ASA, Corticosteroids
125 F	UC	55	F	HF	0	
125 S	UC	55	F	R	0	
126 S	UC	20	M	S	2	5-ASAS/S-ASA, Corticosteroids
127 F NY	CD	38	M	HF	13	5-ASAS/S-ASA, Corticosteroids

128 F	CD	55	M	HF	10	5-ASAS/S-ASA
131 F	N	48	F	HF	10	
132 S	UC	22	M	R	0.5	5-ASAS/S-ASA
133 S	UC	37	F	S	10	
134 S	CD	36	M	S	12	5-ASAS/S-ASA, Corticosteroids
137 F	N	57	M	HF	NA	
138 S	UC	19	M	HF	0.5	5-ASAS/S-ASA
140 F	N	68	M	HF	NA	
141 F	UC	32	M	HF	7	5-ASAS/S-ASA
143 F	N	35	F	HF	NA	
144 F	UC	60	F	HF	25	5-ASAS/S-ASA
146F	UC	69	F	HF	NA	5-ASAS/S-ASA, Corticosteroids
148 S	UC	42	M	T	9	
149 F	UC	59	F	HF	13	5-ASAS/S-ASA
149 S	UC	59	F	R	13	5-ASAS/S-ASA
150 F	CD	50	F	HF	18	
151 S	UC	48	F	R	4	5-ASAS/S-ASA
152 F	UC	38	F	HF	3	Corticosteroids
152 S	UC	38	F	R	3	Corticosteroids
153 S	UC	28	F	S	6	5-ASAS/S-ASA, Corticosteroids
154 F	CD	26	M	HF	0.25	
154 S	CD	26	M	S		
155 S	UC	53	M	S	16	5-ASAS/S-ASA, Corticosteroids
157 F	UC	37	F	R	8	
157 S	UC	37	F	HF	8	
158 F	UC	50	M	HF	8	5-ASAS/S-ASA
159 F	UC	34	M	HF	12	5-ASAS/S-ASA, Corticosteroids
160 F	UC	27	M	HF	23	
161 F	CD	47	M	HF	6	
163 F	UC	21	F	HF	2	
164 S	CD	36	M	C	7	
165 F	UC	39	F	HF	7	5-ASAS/S-ASA
166 S	UC	46	F	R	21	5-ASAS/S-ASA
168 F	UC	57	M	HF	26	5-ASAS/S-ASA

169 F	CD	22	M	HF	0.25	
170 F	UC	59	M	HF	22	5-ASAS/S-ASA
171 F	UC	49	M	HF	22	5-ASAS/S-ASA, Corticosteroids
171 S	UC	49	M	S	22	5-ASAS/S-ASA, Corticosteroids
173 F NY	UC	28	F	HF	8	5-ASAS/S-ASA
173 S NY	UC	28	F	R	8	5-ASAS/S-ASA
175 F	UC	49	F	HF	34	5-ASAS/S-ASA
176 F	UC	31	M	HF	0	
177 F	CD	55	F	HF	2	Corticosteroids
178 F	UC	44	F	HF	15	5-ASAS/S-ASA
180 F	UC	44	F	HF	14	
181 S	UC	72	M	S	15	5-ASAS/S-ASA
182 F	CD	40	M	HF	3	5-ASAS/S-ASA
183 F	UC	49	M	HF	25	5-ASAS/S-ASA
184 F	UC	46	F	HF	0	
200 F NY	N	30	F	HF	NA	
201 F	UC	45	M	HF	12	5-ASAS/S-ASA
202 F	N	28	M	HF	NA	
203 F	N	48	F	HF	NA	
205 F	N	51	M	HF	NA	
206 F	UC	71	F	HF	17	
208 F	UC	62	F	HF	25	
209 F	UC	59	F	HF	40	5-ASAS/S-ASA
209 S	UC	59	F	S	40	5-ASAS/S-ASA
210 F	UC	37	M	HF	19	
210 S	UC	37	M	R	19	
211 F	CD	20	F	HF	0.5	
212 F	CD	42	M	HF	17	5-ASAS/S-ASA
212 S	CD	42	M	S	17	5-ASAS/S-ASA
214 F	UC	33	F	HF	2	
214 S	UC	33	F	R	2	
215 F	UC	56	M	HF	4	5-ASAS/S-ASA
216 F	N	60	M	HF	NA	
217 F	N	42	M	HF	NA	
218 F	CD	49	M	HF	28	
219 F	N	44	F	HF	NA	
222 F	N	35	M	HF	NA	

223 F	N	31	F	HF	NA	
225 F	UC	42	F	HF	15	5-ASAS/S-ASA
225 S	UC	42	F	S	15	5-ASAS/S-ASA
227 S	UC	25	M	R	2	5-ASAS/S-ASA, Corticosteroids
228 F	UC	55	M	T	27	5-ASAS/S-ASA
228 S	UC	55	M	A	27	5-ASAS/S-ASA
229S	CD	31	F	T	7	
230 F	CD	39	M	HF	25	5-ASAS/S-ASA, Corticosteroids
231 F	UC	33	F	HF	4	
233 F	UC	59	F	HF	18	5-ASAS/S-ASA
234 F	N	26	F	HF	NA	
235 F	UC	22	M	HF	1	5-ASAS/S-ASA
235 S	UC	22	M	S	1	5-ASAS/S-ASA
236 F	CD	26	M	HF	8	
239 S	UC	31	F	S	14	5-ASAS/S-ASA
240 F	CD	63	M	HF	6	Corticosteroids
241 F	UC	30	M	HF	10	
242 F	N	30	M	HF	NA	
243 S	UC	35	M	R	15	5-ASAS/S-ASA, Corticosteroids
244 F	UC	62	M	HF	19	5-ASAS/S-ASA
245F	CD	44	F	HF	7	
247F	N	19	F	HF	NA	
250 F	CD	21	M	HF	5	5-ASAS/S-ASA, Corticosteroids
253 F	UC	48	F	HF	17	
254 S	UC	64	F	R	27	5-ASAS/S-ASA
256 F	UC	59	F	HF	20	5-ASAS/S-ASA
257 S	UC	29	F	HF	13	
258 S	UC	32	M	HF	20	5-ASA
259 F	CD	28	F	HF	6	
259 S	CD	28	F	R	6	

Table 1: Sample information for all samples used in the final analysis. Where information on time of first diagnosis is missing, "NA" is given. Sample area is given as follows: R - Rectum, S – Sigmoid colon, D – Descending colon, T – Transverse colon, H – Hepatic flexure, A – Ascending colon, C – Caecum.
